# *SOX1* and *PAX1* Are Hypermethylated in Cervical Adenocarcinoma and Associated with Better Prognosis

**DOI:** 10.1155/2020/3981529

**Published:** 2020-12-07

**Authors:** Zitong Zhao, Xiaoye Zhang, Xueheng Zhao, Jingting Cai, Na-Yi Yuan Wu, Jing Wang

**Affiliations:** Hunan Cancer Hospital, The Affiliated Cancer Hospital of Xiangya School of Medicine, Central South University, China

## Abstract

**Background:**

The increased risk and poor survival outcome of cervical adenocarcinoma (CAC) demand for effective early diagnostic biomarkers that can predict the disease progression and outcome. The purpose of this study was to investigate the value of methylation status of *SOX1* and *PAX1* in the detection and prognosis of CAC.

**Methods:**

We performed a quantitative methylation-specific polymerase chain reaction in 205 cervical paraffin-embedded specimens (175 CACs, 30 noncancer cervical tissues). Overall and progression-free survival (OS and PFS, respectively) rates were calculated and compared using the Kaplan-Meier method. The prognostic value of *SOX1^m^* and *PAX1^m^* on CAC patients was assessed by the Cox regression model. A mathematical formula combining *SOX1^m^*, *PAX1^m^*, and age was constructed for survival prediction.

**Results:**

The methylation status of *SOX1* and *PAX1* was higher in CAC tissues than in noncancer cervical tissues. In addition, *SOX1^m^*-positive CAC patients showed a higher 5-year OS rate than *SOX1^m^*-negative patients. In CAC patients with smaller tumor size (<4 cm), the *PAX1^m^*-positive group showed a higher 5-year PFS rate than the *PAX1^m^*-negative group. In the algorithm combining *SOX1^m^*, *PAX1^m^*, and age, the low-risk group showed a better 5-year OS and PFS rate than the high-risk group.

**Conclusion:**

*SOX1* and *PAX1* methylation levels are higher in CAC than in normal cervical tissues and are potential biomarkers for monitoring CAC prognosis.

## 1. Introduction

Cervical cancer is the second commonest tumor in women worldwide [1]. Moreover, it is a major cause of cancer-related death among women in developing countries [2]. Cervical adenocarcinoma (CAC) ranks second after cervical squamous cell carcinoma (SCC) as the most common pathological type. Although the use of human papillomavirus (HPV) vaccines and effective Pap smear screening have significantly decreased the incidence rate of cervical carcinoma in most regions, the percentage of CAC has been increasing, especially in younger women, accounting for 20-25% of all cervical carcinoma in some developed countries [2, 3]. Moreover, the high propensity of CAC for ovarian metastases always leads to fertility destruction in young women [4–10]. Several studies have shown that at the same stage, CAC has a worse prognosis than SCC, mainly because of its higher rate of metastase [6] and lower sensitivity to radiotherapy and chemotherapy [11]. The increased frequency and poor survival outcome raise the need for useful biomarkers that can predict the progression and prognosis of CAC.

Epigenetic alterations include heritable DNA methylation and histone protein modifications that do not affect the DNA transcriptional sequence [12–15]. DNA methylation is an epigenetic alteration, which always occurs in the early stage of carcinogenesis, leading to lessen even lost expression of the methylated gene [16]. Expression of *SOX1* correlated with early embryogenesis, central nervous system development, and neural stem cell maintenance [17]. Nonetheless, hypermethylation in the promoter region and/or somatic mutations in the so-called tumor suppressor genes might cause silencing or inhibition of *SOX1*, which in turn may result in cancer cell proliferation and migration and finally progression of cervical carcinogenesis [18]. Hypermethylated *SOX1* (*SOX1^m^*) has been associated with several cancer types, including hepatocellular cancer, lung cancer, urothelial bladder cancer, endometrial cancer, and SCC [19–22]. *PAX1* expression is correlated with embryogenesis, particularly with the development of the thymus, parathyroid glands, and skeleton [23–25]. Hypermethylated *PAX1* (*PAX1^m^*) has been found in ovarian cancer, oral cancer, and SCC [26–29]. Our previous study confirmed *SOX1* and *PAX1* methylation as promising screening biomarkers in cervical neoplasia, mainly in high-grade squamous intraepithelial lesions and SCC, because of its remarkable discriminating ability between normal tissues and high-grade cervical lesions [30–33]. However, CAC and SCC are different with respect to tumor development, progression, and molecular pathology. It remains unknown whether the methylation status of *SOX1* and *PAX1* is different between CAC and SCC. In addition, the methylation level and prognostic value of *SOX1* and *PAX1* for CAC are unclear. In this study, we investigated the methylation levels of *SOX1* and *PAX1* differ in CAC and evaluated the potential value of *SOX1* and *PAX1* gene methylation for prognosis in CAC.

## 2. Methods

### 2.1. Patients and Tissue Specimens

A total of 205 cervical paraffin-embedded specimens between 2013 and 2015 were collected from the Hunan Cancer Hospital, the Affiliated Cancer Hospital of Xiangya School of Medicine, Central South University, including 30 noncancer cervical samples and 175 adenocarcinoma samples (Table 1). The patients' demographic and clinicopathological data, including clinical stage (FIGO Committee on Gynecologic Oncology, 2009), tumor size, depth of invasion, histologic tumor grade, lymph node metastasis, and treatment modality, were recorded. The study protocols were agreed by the Hunan Cancer Hospital ethics committee (project number: 2015[01]) and the Chinese Clinical Trial Registry (registration number: ChiCTR1800018931).

### 2.2. DNA Preparation, Bisulfite Conversion, and Quantitative Methylation-Specific Polymerase Chain Reaction (qMSP)

An ISO17025-certified laboratory (iStat Biomedical Co., Ltd., New Taipei City, Taiwan) carried out total methylation tests. They first deparaffinized paraffin-embedded cervical tissues and then extracted genomic DNA (gDNA) samples and bisulfite converted by using an Epigene™ nucleic acid extraction kit and an Epigene™ bisulfite conversion kit (iStat Biomedical Co., Ltd., New Taipei City, Taiwan). Quantitative methylation-specific PCR (qMSP) was then used for analyzing the methylation level of *SOX1* and *PAX1* by the TaqMan Probe system in a Light Cycler LC480 system (Roche Applied Science, Penzberg, Germany). Our previous study described specific primers and probes for qMSP [31, 34, 35]. The registered A375 and CaSki two cancer cell lines were treated as methylation and nonmethylation controls to ensure the quality of the bisulfite conversion and qMSP processes. The DNA methylation level was assessed as the methylation index (M-index) using the formula [36]: 10,000 × 2^(Cpvalueofgene − Cpvalueof*COL*2*A*)^. *SOX1* and *PAX1* (positive) were deemed to be hypermethylated (positive) if the delta Cp was smaller than 11 and 9, respectively.

### 2.3. Statistical Analyses

The cut-off values for *SOX1^m^* and *PAX1^m^* were generated from 205 clinical samples (175 CACs and 30 noncancer cervical tissues). Receiver operating characteristic (ROC) curves were performed, and the area under the ROC curve (AUC) was calculated for the detection of the CAC.

All statistical analyses were performed using GraphPad Prism® 7.00 (GraphPad, La Jolla, CA, USA) and SPSS Statistics 24 (SPSS, Inc., Chicago, IL, USA). The correlation between *SOX1^m^* or *PAX1^m^* and each clinicopathological characteristic of the CAC patients was evaluated by the Mann-Whitney and Dunnett's tests. Kaplan-Meier method was used to describe the progression-free survival and overall survival (PFS and OS). The PFS was judged from treatment to the date of the first relapse at any site or death including all causes, and OS was calculated from treatment to death covering any cause. Hazard ratio (HRs) was calculated with multivariate Cox regression analysis.

### 2.4. A Mathematical Algorithm Combining *SOX1^m^*, *PAX1^m^*, and Age for CAC Prognosis Prediction

To investigate the effectiveness of the combination of the methylation statuses of these two genes and the clinicopathological factors to predict the clinical outcome, we constructed a mathematical formula for survival prediction. Each patient was assigned with a risk score in accordance with a linear combination of the expression level of the two genes. The risk score was calculated as follows: risk score = [*W*1 × *SOX*1^*m*^] + [*W*2 × *PAX*1^*m*^] + [*W*3 × age]. The weight factors (*W1–3*) were generated from the regression coefficients derived from the aforementioned-univariate Cox regression analysis (Lossos et al., 2004). We divided patients into low-risk and high-risk groups according to the median risk score as the cut-off point. The OS and PFS were then estimated by the Kaplan-Meier method.

## 3. Results

### 3.1. *SOX1* and *PAX1* Are Hypermethylated in CAC Tissues than in Noncancer Cervical Tissues

**Figure 1:**
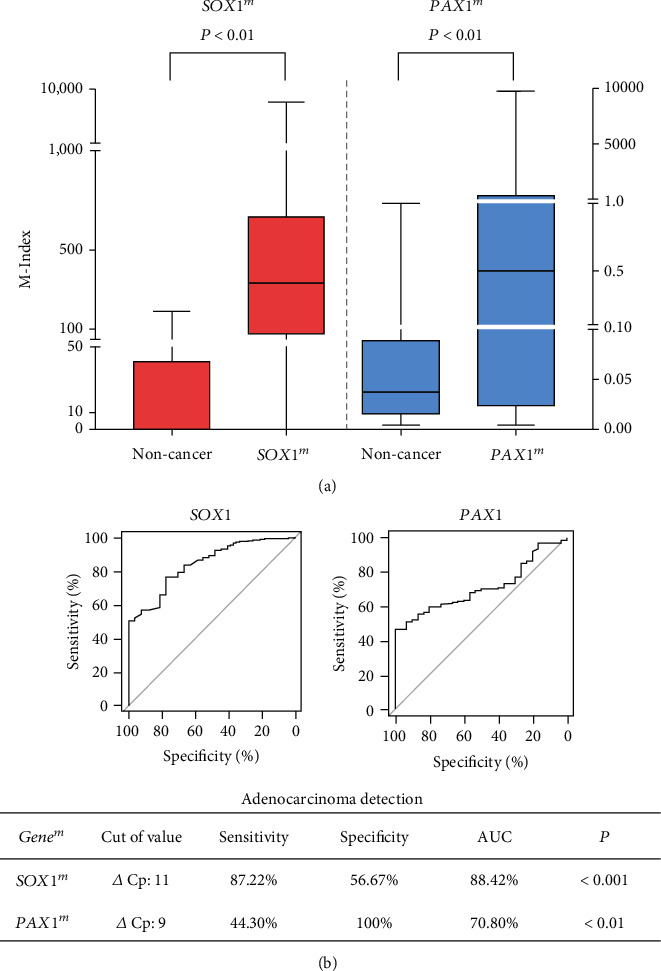
*SOX1* and *PAX1* methylation levels in cervical adenocarcinoma. (a) Methylation-Index (M-Index) of *SOX1* and *PAX1* methylation between the noncancer group (30 noncancer tissues) and cervical cancer group (175 adenocarcinoma), *p* < 0.01; (b) the area under the ROC curve for the *SOX1* and *PAX1* methylation assay was calculated for exploring cervical cancer. The sensitivity and specificity of *SOX1* methylation were 87.22% and 56.67%, respectively, with a cut-off point of *∆*Cp = 11. The AUC was 88.42%. And the sensitivity and specificity of *PAX1* methylation were 40.30% and 100%, separately, with a cut-off point of *∆*Cp = 9. The AUC was 70.80%.

Among our 205 cervical specimens, 30 were noncancer cervical samples and 175 were adenocarcinoma samples (Table 1). The mean M-index for SOX1 (476.80 ± 92.47 and 0.48 ± 0.29, respectively, *p* < 0.05) and PAX1 (515.70 ± 56.30 and 28.19 ± 9.19, respectively, *p* < 0.05) was significantly higher in CAC tissues than in noncancer tissues (Figure 1(a)). By ROC analysis, the positive cut-off value for *SOX1^m^* was *∆*Cp ≤ 11, with a high AUC level of 88.42%, a sensitivity of 87.22%, and specificity of 56.67%. The positive cut-off value for *PAX1^m^* was *∆*Cp ≤ 9, with a high AUC level of 70.80%, sensitivity of 44.30%, and specificity of 100% (Figure 1(b)), which suggested that *SOX1^m^* and *PAX1^m^* may be detection biomarkers for CAC.

The *SOX1* and *PAX1* methylation statuses showed no significant difference based on age, FIGO stage, tumor size, depth of invasion, lymph node metastasis, and histologic grade in CAC patients (Table 2).

### 3.2. Hypermethylated *SOX1* and *PAX1* Are Associated with Better Survival in CAC Patients

**Figure 2:**
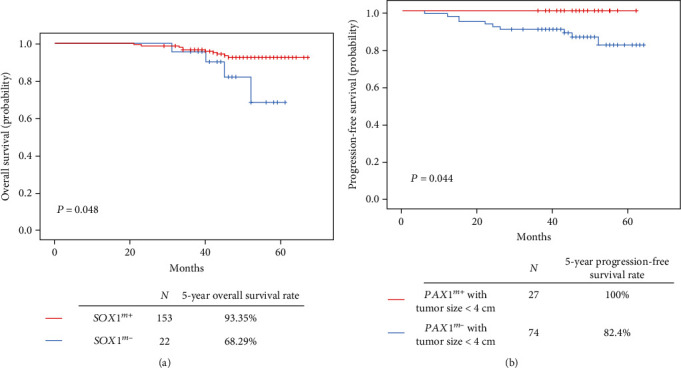
Association of *SOX1* and *PAX1* methylation status with CAC patients' survival. (a) *SOX1^m^*-positive patients have a longer OS rate than *SOX1^m^*-negative patients (93.35% vs. 68.29%, *p* = 0.048); (b) in CAC patients with smaller tumor size (<4 cm), *PAX1^m^*-positive group had a higher 5-year PFS rate than the *PAX1^m^*-negative group (100% vs. 82.4%, *p* = 0.044).

Further studies were conducted to investigate whether the methylation of *SOX1* and *PAX1* was correlated with the prognosis in CAC patients. The *SOX1^m^*-positive group showed a higher 5-year OS rate of 93.35% than the *SOX1^m^*-negative group, which showed a 5-year OS rate of 68.29% (*p* = 0.048) (Figure 2(a)). While no remarkable finding was obtained in the analysis of the 5-year PFS rate. For *PAX1*, there was no evident difference in 5-year OS rate or 5-year PFS rate between these two groups (data not shown). However, in CAC patients with smaller tumor size (<4 cm), the *PAX1^m^*-positive group had a higher 5-year PFS rate than the *PAX1^m^*-negative group (100% vs. 82.4%, *p* = 0.044) (Figure 2(b)).

### 3.3. Algorithm Combining *SOX1^m^*, *PAX1^m^*, and Age for Prognosis of CAC Patients

**Figure 3:**
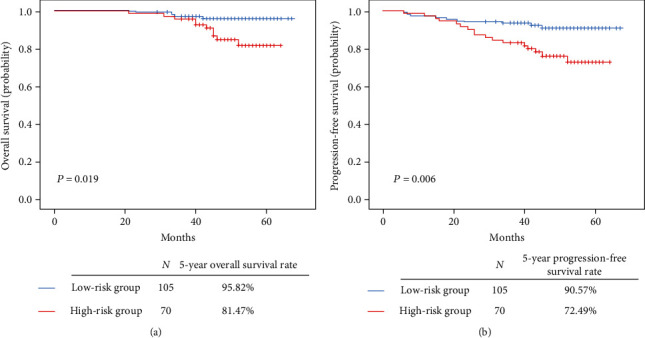
Algorithm combining clinicopathologic factor and genetic test results. The algorithm divided patients into high-risk and low-risk groups. The low-risk group showed a better 5-year overall survival (OS) rate (95.89% vs. 81.47%, *p* = 0.019) (a) and better 5-year progression-free survival (PFS) rate (90.58% vs. 72.50%, *p* = 0.006) than the high-risk group (b).

We established an algorithm for quantifying age, *SOX1^m^*, and *PAX1^m^* as a prognostic factor to calculate the recurrence risk and outcome of patients with CAC. The final algorithm for OS was as follows: risk score = [−1.109 × *SOX*1^*m*^] + [−0.849 × *PAX*1^*m*^] + [0.399 × age]. The final algorithm for PFS was as follows: risk score = [−0.586 × *SOX*1^*m*^] + [−0.553 × *PAX*1^*m*^] + [0.674 × age]. The algorithm divided patients into low-risk and high-risk groups. However, the low-risk group showed a better 5-year OS rate (95.89% vs. 81.47%, *p* = 0.019) (Figure 3(a)) and a much better 5-year PFS rate (90.58% vs. 72.50%, *p* = 0.006) than the high-risk group (Figure 3(b)).

## 4. Discussion

It is well known that the prognosis of CAC is worse than cervical SCC, even for early-stage patients, especially in developing countries [37]. Gene methylation is an epigenetic modification, which has tumor-suppressive or tumorigenic two opposite effects, possibly playing key roles in carcinogenesis and cancer progression. Before this study, the methylation status and prognostic value of *SOX1^m^* and *PAX1^m^* for CAC were unclear. In this study, we observed higher methylation levels of *SOX1* and *PAX1* in CAC than in noncancer cervical tissues. For the detection of CAC, *SOX1^m^* showed 87.22% sensitivity and 56.67% specificity, while *PAX1^m^* showed 44.30% sensitivity and 100% specificity. Moreover, several studies have demonstrated that *PAX1* methylation increases following increased disease grade: *PAX*1 methylation in SCC > high − grade squamous intraepithelial lesion (HSIL) > low − grade squamous intraepithelial lesion (LSIL) > normal tissue [38, 39]. These suggest that hypermethylation of *SOX1* and *PAX1* might play an important role in the diagnosis and cancer progression of CAC.

The current study also showed, for the first time, that CAC patients who are *SOX1^m^*-positive show a better prognosis, suggesting that *SOX1* gene methylation has the potential to predict the 5-year OS. In CAC patients with small tumor size (<4 cm), *PAX1^m^*-positive patients showed a longer 5-year PFS, suggesting that *PAX1* gene methylation might be useful for monitoring the 5-year progression in CAC patients with small tumor size. This study, meanwhile, has several limitations. First, our patients all came from a single medical center and the sample size was comparatively small. Second, the detailed molecular relationship between *SOX1^m^* and *PAX1^m^* in CAC has not been explored, which deserves further investigation.

Despite the poor prognosis of CAC, there is still no valid prognostic risk model. By far, age is a strong risk factor for cancer [40, 41]. The current paper showed corresponding directed changes in DNA methylation with age, which is characterized by hypermethylation of targets of polycomb group proteins (PCGTs) that are crucial in embryonic stem cell lineage differentiation [42]. In the algorithm combining *SOX1^m^*, *PAX1^m^*, and age, the low-risk group, composed of high methylation level of *SOX1* and *PAX1* and younger age, showed a significantly higher 5-year OS rate and 5-year PFS rate than the high-risk group. These results suggested that the algorithm has the potential for a 5-year CAC prognosis.

In our previous study, negative gene methylation correlated with high protein expression, which increased the resistance of cervical cancer cells to radiation and chemotherapy [43]. In further studies, it will be essential to analyze the correlation between *SOX1* and *PAX1* methylation status and sensitivity of the cervical cancer cell to radiotherapy and chemotherapy.

In conclusion, this study suggests that *SOX1* and *PAX1* methylation levels are higher in CAC than in cervical SCC, and *SOX1^m^* and *PAX1^m^* are potential biomarkers for monitoring CAC prognosis.

## Figures and Tables

**Table 1:** Clinicopathologic characteristics of 205 patients.

Characteristics	Numbers (%)
Specimens (*N* = 205)	
Adenocarcinoma	175 (85.37%)
Noncancer tissues	30 (14.63%)
Cancer group (*N* = 175)	
Age (years)	
<50	96 (54.86%)
≥50	79 (45.14%)
FIGO stage	
<IIB	127 (71.57%)
≥IIB	48 (27.43%)
Tumor size	
<4	101 (57.71%)
≥4	72 (41.14%)
Deep of invasion	
<1/2	74 (42.29%)
≥1/2	90 (51.43%)
LNM	
No	126 (72%)
Yes	41 (23.43%)
Histologic grade	
Well/moderate	119 (68%)
Poor	45 (25.71%)

Abbreviations: FIGO: International Federation of Gynecology and Obstetrics; LNM: lymph node metastasis.

**Table 2:** Association between *SOX1m* or *PAX1m* and clinicopathological characteristics in 175 CAC patients.

Variable	Number of patients	*SOX1* ^*m*^			*PAX1* ^*m*^		
	(*n* = 175)	Positive	Negative	*p* value	Positive	Negative	*p* value
Age (years)				0.625			0.91
<50	96 (54.86%)	83	13		28	68	
≥50	79 (45.14%)	70	9				
FIGO stage				0.937			0.766
<IIB	127 (71.57%)	112	15		36	91	
≥IIB	48 (27.43%)	41	6		14	33	
Tumor size				0.748			0.412
<4	101 (57.71%)	89	12		27	74	
≥4	72 (41.14%)	62	10		23	49	
Deep of invasion				0.929			0.863
<1/2	74 (42.29%)	64	10		21	53	
≥1/2	90 (51.43%)	78	12		24	66	
LNM				0.418			0.17
No	126 (72%)	111	15		37	89	
Yes	41 (23.43%)	34	7		8	33	
Histologic grade				0.644			0.146
Well/moderate	119 (68%)	104	15		33	86	
Poor	45 (25.71%)	38	7		17	28	

## Data Availability

The data used to support the findings of this study are included in the article.

## Abbreviations

CAC: Cervical adenocarcinoma
SCC: Cervical squamous cell carcinoma
SOX1^m^: Methylation status of SOX1
PAX1^m^: Methylation status of PAX1
FIGO: International Federation of Gynecology and Obstetrics
PFS: Progression-free survival
OS: Overall survival.
